# Effects of a polysaccharide-rich extract derived from Irish-sourced *Laminaria digitata* on the composition and metabolic activity of the human gut microbiota using an in vitro colonic model

**DOI:** 10.1007/s00394-019-01909-6

**Published:** 2019-02-25

**Authors:** Conall R. Strain, Kenneth C. Collins, Violetta Naughton, Emeir M. McSorley, Catherine Stanton, Thomas J. Smyth, Anna Soler-Vila, Mary C. Rea, Paul R. Ross, Paul Cherry, Philip J. Allsopp

**Affiliations:** 1 Nutrition Innovation Centre for Food and Health (NICHE), Ulster University, Coleraine, BT52 1SA UK; 2 Teagasc Food Research Centre, Moorepark, Fermoy, Ireland; 3 Department of Life Science, Institute of Technology Sligo, Sligo, Ireland; 4 Irish Seaweed Research Group, Ryan Institute for Environmental, Marine and Energy Research, National University of Ireland, Galway, University Road, Galway, Ireland; 5 APC Microbiome Institute, University College Cork, Cork, Ireland

**Keywords:** Seaweeds, Macroalgae, *Laminaria digitata*, Dietary fiber, Prebiotic, Gut microbiota, Short-chain fatty acids, Metagenomics

## Abstract

**Background:**

Brown seaweeds are known to be a rich source of fiber with the presence of several non-digestible polysaccharides including laminarin, fucoidan and alginate. These individual polysaccharides have previously been shown to favorably alter the gut microbiota composition and activity albeit the effect of the collective brown seaweed fiber component on the microbiota remains to be determined.

**Methods:**

This study investigated the effect of a crude polysaccharide-rich extract obtained from *Laminaria digitata* (CE) and a depolymerized CE extract (DE) on the gut microbiota composition and metabolism using an in vitro fecal batch culture model though metagenomic compositional analysis using 16S rRNA FLX amplicon pyrosequencing and short-chain fatty acid (SCFA) analysis using GC-FID.

**Results:**

Selective culture analysis showed no significant changes in cultured lactobacilli or bifidobacteria between the CE or DE and the cellulose-negative control at any time point measured (0, 5, 10, 24, 36, 48 h). Following metagenomic analysis, the CE and DE significantly altered the relative abundance of several families including *Lachnospiraceae* and genera including *Streptococcus, Ruminococcus* and *Parabacteroides* of human fecal bacterial populations in comparison to cellulose after 24 h. The concentrations of acetic acid, propionic acid, butyric acid and total SCFA were significantly higher for both the CE and DE compared to cellulose after 10, 24, 36 and 48 h fermentation (*p* < 0.05). Furthermore, the acetate:propionate ratio was significantly reduced (*p* < 0.05) for both CD and DE following 24, 36 and 48 h fermentation.

**Conclusion:**

The microbiota-associated metabolic and compositional changes noted provide initial indication of putative beneficial health benefits of *L. digitata* in vitro; however, research is needed to clarify if *L. digitata*-derived fiber can favorably alter the gut microbiota and confer health benefits in vivo.

## Introduction

Macroalgae as a food resource remains a substantial component of the human diet with over 2 million kg of edible seaweed harvested globally every year [1]. There is increasing interest in the health benefits associated with seaweed consumption, particularly in relation to the reduced risk of age-associated chronic diseases in Japanese populations; where seaweed consumption has been estimated to be as high as 5.3 g/day [2]. The high fiber content of seaweed, up to 46 g/100 g semi-dry sample weight [3], has been suggested to contribute to the health benefits associated with seaweed consumption [4].

The health benefits of dietary fiber have been widely recognized, where a daily intake of 25 g/day and up to 38 g/day has been recommended by the European Food Safety Authority and US Institute of Medicine, respectively [5, 6], owing to empirical evidence for dietary fiber in reducing the risk of obesity, type 2 diabetes, and coronary heart disease [7]. The mechanisms to support a role for fiber in health have been associated with its viscosity and fecal bulking properties which may impact the rate and nature of gastrointestinal digestion/absorption and transit time [8]. There is also considerable evidence to support a possible role for fiber in promoting health through its ability to modulate gut microbiota composition and metabolism [9]. The proposed benefits of fiber on the intestinal microbiota are associated with their uptake and utilization by putative health-promoting bacteria species and the subsequent cross-species metabolism of fermentation by-products [10–13]. The short-chain fatty acid (SCFA) microbial metabolites are of particular interest, with suggestions that they can promote health through regulation of gut hormone release, cholesterol synthesis/metabolism, enhanced satiety as well as exerting anticancer and anti-inflammatory effects [14–16]. Each fiber source will exert different effects on the microbiota as the fermentation properties of non-digestible polysaccharides are strongly influenced by the monomer composition, type of bond, and degree of polymerization (DP) [9, 17].

The majority of research investigating the health benefits associated with fiber and/or its fermentation properties have predominantly focused on cereal and vegetable derived fiber [18]. More recently, there is increasing interest in the putative health benefits that may be associated with the fermentation of brown seaweed fiber owing to the presence of polysaccharides not typically present in high amounts in the human diet, including alginates (blocks of 1–4-linked β-mannuronate and α-guluronate glycosides), fucoidan (primarily made up of sulphated 1,2- 1,3- and 1,4-linked α-l-fucose) and laminarins (a 1–3-linked β-glucan backbone with short 1–6 β-linked side chains) [19–22]. A small number of studies have investigated the effects of the individual polysaccharides derived from brown seaweed on the gut microbiota composition using both in vitro and in vivo methods [23–35]. These studies have provided initial indications of the potential fermentability of these brown seaweed fiber components by the human microbiota. Nevertheless, it must be noted that the fermentation and metabolism of each individual non-digestible polysaccharide are considerably influenced by the availability of other, potentially competing, polysaccharide substrates that may be present in the digesta. In summary, extrapolating the putative health benefits of seaweed fiber from the aforementioned studies which investigated singular polysaccharides is limited by the absence of simultaneous exposure to the numerous polysaccharides present in the seaweed. This study aimed to investigate the fermentation properties of a relatively crude fiber mixture derived from the brown seaweed, *Laminaria digitata*, in an attempt to determine the fermentation properties of the whole fiber component. This study specifically evaluated the effects of a crude polysaccharide-rich extract obtained from *L. digitata*, (CE), and a processed depolymerized CE (DE), both of which were previously subjected to a simulated in vitro digestion, on the microbial composition and metabolic activity of the human gut microbiota, where fructooligosaccharides (FOS) and cellulose served as positive and negative controls, respectively, using an in vitro human colonic model.

## Materials and methods

### Harvesting of *L. digitata*

*Laminaria digitata* was harvested at Finavarra, County Clare (53.159294, − 9.100080), during low tide (12.30 p.m.–4.00 p.m.) one day in June 2012. Identification of macroalgae species was carried out on-site by a marine biologist (National University of Ireland, Galway). The seaweed was gathered by detaching the holdfast from the rock using a knife, placed in black refuse bags, and transferred within 6 h to storage at − 20 °C until processing.

### Polysaccharide extraction

#### Crude seaweed powder production

The seaweed was washed with freshwater to remove sand and epiphytes and laid onto trays and blast frozen (Blast freezer, New-Avon). The frozen seaweed was freeze-dried (FD80, Cuddon Freeze Dry, New Zealand) and processed into a powder using a Waring blender (New Hartford, CT, USA) and stored in vacuum-packed bags at − 20 °C prior to extraction.

#### Hot acid extraction

Ground seaweed was suspended in 0.1 M HCl at a ratio of 1:10 (w/v). The extractions were carried out by incubating in an orbital shaker (MaxQ 6000 Shaker; Thermo Fisher Scientific, Dublin, Ireland) at 70 °C at 175 rpm for three successive time periods of 3, 3 and 24 h. The extracts were filtered with a muslin bag with the extract removed after each period and fresh solvent added to the retentate. The three extract batches were collated and underwent further filtering with cotton and glass wool using a funnel, Buchner flask and vacuum pump. The filtrates were neutralized with the addition of 20 M NaOH and freeze-dried (FD80, Cuddon Freeze Dry, New Zealand).

### Purification and further processing of extract

#### Ethanol precipitation

The freeze-dried acid extract was dissolved in Milli-Q water (1:2 w/v). This solution was mixed with analytical-grade ethanol (1:5 v/v) before centrifugation at 8000*g* for 10 min. The supernatant was subsequently discarded and the pellet obtained was allowed to air dry overnight in a fume cabinet before blast freezing and freeze-dried (FD80, Cuddon Freeze Dry, New Zealand) to produce the crude extract (CE).

#### Soluble and insoluble dietary fiber analyses

Soluble dietary fiber (SDF) and insoluble dietary fiber (IDF) were measured according to AOAC 991.43, AACC 32-07.01, NMKL 129, 2003 methods using the ANKOM Dietary Fiber analyser (Macedon NY, USA).

#### Depolymerization of extract

A sample of CE underwent a depolymerization step using a Fenton’s reaction. CE was dissolved in 0.04% FeSO_4_ (1:5 w/v) solution to which H_2_O_2_ (30% puriss grade) was added (1:40 v/v). The flask was incubated in an orbital shaker (MaxQ 6000 Shaker; Thermo Fisher Scientific, Dublin, Ireland) for 45 min at 80 °C and 175 rpm. The solution was then blast frozen and freeze-dried (FD80, Cuddon Freeze Dry, New Zealand) to produce the depolymerized CE extract (DE).

#### Simulated human gastrointestinal digestion

Prior to carrying out the in vitro digestion both the CE and DE underwent size-exclusion dialysis using dialysis tubing with a molecular weight cutoff of 1 kDa (Standard RC dialysis tubing, Spectrum labs, Rancho Dominguez, USA). For this, the extracts were dissolved in Milli-Q water (1:1 w/v) and placed within the dialysis tubing. The tubes were closed with clips and placed in 10 L of Milli-Q water and incubated in an orbital shaker (MaxQ 6000 Shaker; Thermo Fisher Scientific, Dublin, Ireland) at 25 °C, 50 rpm for 72 h. The water was changed every 24 h throughout the dialysis. The dialysate was discarded and the remaining extract was freeze-dried (FD80, Cuddon Freeze Dry, New Zealand). 60 g of each sample obtained (> 1 kDa CE and DE, and cellulose) was suspended in 180 mL of Milli-Q water (1:4 w/v) and added to a 500-mL Erlenmeyer flask to which a 6.25 mL solution of CaCl_2_ (1 mM pH 7) with 20 mg of dissolved α-amylase had been added. The solutions were incubated in an orbital shaker (MaxQ 6000 Shaker; Thermo Fisher Scientific, Dublin, Ireland) at 150 rpm at 37 °C for 30 min. On completion of the incubation, the solutions were adjusted to pH 2 with concentrated HCl (37% puriss, 12.06 M). A pepsin solution of (2.7 g pepsin in 125 mL of 0.1 M HCl) was then added to each sample and the sample solutions were incubated in an orbital shaker (MaxQ 6000 Shaker) at 37 °C 150 rpm for 2 h. The solutions were neutralized with 1 M NaOH and 125 mL of 0.5 M NaHCO_3_ followed by the addition of 560 mg pancreatin and 3.5 g bile extract. The solutions were adjusted to pH 7 with either the addition of 1 M HCl or 1 M NaOH and further incubated in an orbital shaker (MaxQ 6000 Shaker) at 37 °C, 150 rpm for 3 h. The sample solutions (CE, DE and Cellulose) were subsequently transferred into 1-kDa dialysis tubing (Standard RC dialysis tubing, Spectrum labs) and placed in Milli-Q water for 72 h with the dialysate replaced every 24 h with fresh Milli-Q water. The solutions were then blast frozen and freeze-dried (FD80, Cuddon Freeze Dry, New Zealand) and stored at − 20 °C until use.

The controls used in the in vitro study were fructooligosaccharides (FOS) and cellulose. FOS was selected as a positive control as it is a prebiotic fiber, while cellulose was chosen as a negative control as it is poorly fermented. The FOS solution did not undergo the in vitro digestion process as it is known to be resistant to digestion and would not be retained by the 1-kDa-molecular weight-cutoff dialysis tubing. To account for any components which may result from the in vitro digestion procedure, an in vitro digestion was carried out without any substrate and the resulting freeze-dried material was added to FOS in the correct ratio.

### Fecal fermentations

#### Basal growth media

The basal growth medium used was similar to Fooks and Gibson [36] [2 g of peptone water (Oxoid thermos Fischer scientific, Renfrew, UK), 2 g of yeast extract (Oxoid thermos Fischer scientific, Renfrew, UK), 0.1 g NaCl, 0.04 g of K_2_HPO_4_, 0.04 g of KH_2_PO_4_, 0.01 g of CaCl_2_·6H_2_0, 0.01 g of MgSO_4_·7H_2_0, 0.2 g of NaHCO_3_, 2 mL of Tween-80 (Sigma-Aldrich, St Louis, USA), 0.05 g of Hemin (Sigma-Aldrich, St Louis, USA) dissolved in 0.5 mL of 1 M NaOH, 10 µL of vitamin K dissolved in 200 µL of ethanol, 0.5 g of cysteine–HCl (Sigma-Aldrich, St Louis, USA), 0.5 g bile salts powder (Oxoid thermos Fischer scientific, Renfrew, UK)]. The pH of the media was reduced to 6.8 by the addition of 1 M HCl, autoclaved, and allowed to cool to 37 °C before use in the experiments.

#### Fecal sample collection and processing

Fecal fermentation experiments were carried out in triplicate on 3 different days. Freshly voided fecal samples from three healthy donors (Male, Caucasian Aged 18–32) were obtained within 1 h prior to the beginning of each experiment. A 20 g portion of fecal sample of each donor was collected in a Stomacher strainer bag (Seward Limited, Worthington, UK) to which 240 mL of autoclaved maximum recovery diluent (MRD, Oxoid Ltd, Hampshire, UK) cysteine–HCl solution was added. The mixture was homogenized using Seward Stomacher 400 circulator machine (Seward Limited, Worthington, UK) at a normal speed for 1 min.

#### Fermentation vessel setup and sampling

The fermentations were carried out in two Multifors units (four vessels in total; Infors, Bottmingen, Switzerland) with 160 mL of basal growth medium added to each vessel. First, the vessels were purged with filtered nitrogen gas for 2 h prior to the addition of the fecal slurry and for the duration of the experiment. After purging, 2 g of the extracts and controls (2%) were subsequently added to their respective fermentation vessel. The vessels were maintained at 37 °C and pH 6.8 with stirrers set at 100 rpm. A 40 mL sample of fecal slurry was immediately injected into each vessel. Within 2 min following the addition of fecal slurry, a 13 mL aliquot was removed from each vessel for analysis to represent a baseline (T0), with further samples collected at 5, 10, 24, 36 and 48 h. From each 13 mL sample collected, 2 × 1 mL aliquots were immediately used for culture-dependent bacterial enumeration. Also, a 5 mL sample was spun at 15,000 rpm for 5 min, and a total of 3 × 1 mL aliquots of supernatant were removed and stored at − 80 °C for future short-chain fatty acid (SCFA) analysis. For culture-independent PCR-based GM analysis, 2 × 1 mL from each sample were aliquoted into microtubes and centrifuged at 15,000 rpm, with the supernatant discarded and pellets stored for pyrosequencing and stored at − 80 °C until analysis.

### Enumeration, isolation and identification of *Bifidobacteria* and *Lactobacillus* species

#### Sample dilution

A 1 mL aliquot of the fermenter slurry sample was added to 9 mL of sterile MRD (Oxoid Ltd). A serial dilution was carried out by removing 1 mL from the previous tube and adding to the next 9 mL of MRD for a total of seven serial dilutions in duplicate.

The dilutions of the samples were then inoculated in duplicate onto selective agars, i.e., *Lactobacillus* selective (LBS) agar (Difco Becton Dickinson, Franklin Lakes, USA) supplemented with acetic acid (32 mM) and MRS agar (Oxoid thermos Fischer scientific, Renfrew, UK) with added 0.2 g of l-cysteine–HCl and 100 × 200-µg mupirocin disks (Oxoid thermos Fischer scientific, Renfrew, UK). Inoculated LBS plates were incubated in an anaerobic cabinet at 37 °C for 120 h while MRS plates were incubated for 72 h before enumerating colonies on a SC6 plus Colony Counter (Stuart equipment, Stone, UK); for confirmation of species, five colonies from each plate were randomly selected and Gram stained.

### Analysis of short-chain fatty acids

#### Internal standard and external standard curve generation

As an internal standard, 2-ethyl butyric acid was added to both samples and standard curve solution at 1 mM. The seven SCFAs (acetic acid, propionic acid, iso-butyric acid, butyric acid, valeric acid, iso-valeric acid and hexanoic acid at 0.1 mM, 0.5 mM, 1 mM, 2 mM, 4 mM, 8 mM and 10 mM) were used to generate a standard curve. Additional 2 mM SCFA standard vials were used to check for reproducibility. A standard curve and five repeat injections of 2 mM SCFA were carried out prior to analysis.

#### Sample processing

A 200 µL sample of fermenter slurry supernatant was added to a 1.5-mL microtube along with 700 µL of Milli-Q water and 100 µL of 10 mM of 2-ethyl butyric acid. The samples were spun at 5,000 rpm and the supernatants were transferred to a 1.5-mL glass vial (Agilent Technologies, Santa Clara, USA).

#### GC-FID analysis

The concentration of SCFA was determined by gas chromatography flame ionization detection (GC-FID) using a Varian 3500 GC system, fitted with a TRB-FFAP column (30 m × 0.32 mm × 0.50 µm; Teknokroma, Barcelona, Spain) and a flame ionization detector. Helium was supplied as the carrier gas at an initial flow rate of 1.3 mL/min. The initial oven temperature was 100 °C, and was maintained for 0.5 min, and then raised to 180 °C at 8 °C/min and held for 1.0 min, before being increased to 200 °C at 20 °C/min, and finally held at 200 °C for 5.0 min. The temperatures of the detector and the injection port were set at 250 °C and 240 °C, respectively. The injected sample volume was 0.5 µL. Peaks were integrated using Varian Star Chromatography Workstation version 6.0 software.

### 454 Pyrosequencing

#### DNA extraction

Total microbial DNA was extracted from pellets of 1 mL of fermenter slurry using a MO BIO PowerFecal DNA isolation kit (Mo Bio, Carlsbad, USA) according to the manufacturer’s instructions. To ensure complete lysis of bacterial cells, an additional heating step was carried out at 90 °C for 10 min prior to the bead beating step. The extracted DNA was quantified in triplicate using a Nanodrop 3300 spectrophotometer (Thermo Scientific, Ireland) and stored at − 80 °C for future PCR amplification of the V4 amplicon.

#### Generation of 16S rDNA amplicons

PCR amplification of the V4 region was carried out using the KAPA3G Plant PCR kit (Kapa Biosystems, USA). Amplicons of the V4 region were generated by employing one forward, i.e., F1 (5′ AYTGGGYDTAAAGNG), and a mix of four reverse primers, i.e., R1 (5′ TACCRGGGTHTCTAATCC), R2 (5′ TACCAGAGTATCTAATTC), R3 (5′ CTACDSRGGTMTCTAATC) and R4 (5′ TACNVGGGTATCTAATC). In addition, the F1 contained an A adaptor sequence along with a distinct multiple identifier (MID) for each sample, while the R primers contained a B adaptor. The PCRs were carried out on a G-storm PCR machine under the following conditions: heated lid 110 °C, 95 °C for 10 min followed by 47 cycles of 95 °C for 20 s, 55 °C for 15 s and 72 °C for 15 s followed by a 1 min at 72 °C after which the samples were held at 4 °C. The PCRs had a final volume of 50 µL and consisted of 25 µL of KAPA3G Plant PCR buffer, 0.4 µL of KAPA3G plant DNA polymerase, 1 µL of F1 primer (0.15 µM), 1 µL of the R1–4 primers (0.15 µM), 1 µL of MgCl2 (to give a final concentration of 2 mM), sterile PCR-grade water and 2.5 µL template DNA. PCR products were analyzed by agarose gel electrophoresis (1.5% in 1× TBE buffer + 2 µL of Midori Green Advance DNA stain (Nippon Genetics)) loading 5 µL of PCR product with 2 µL of Bioline DNA loading buffer, blue. The remaining PCR product (45 µL) of each sample was cleaned using Agentcourt AMPure XP kit (Beckman Coulter Genomics, UK) as per the manufacturer’s protocol. The cleaned PCR products were eluted in 40 µL of solution C6 from the PowerFecal kit. The pooled sample underwent further cleaning using the AMPure XP kit and then quantified again with the Quant-iT Picogreen kit. Emulsion-based clonal amplification was carried out before sequencing on a Genome Sequencer FLX platform (Roche Diagnostics Ltd, West Sussex, UK) according to the manufacturer’s protocols. Sequencing was carried out at the Teagasc Moorepark Sequencing facility.

#### Bioinformatics analysis

Quality trimming of raw sequences was carried out using the QIIME program suite [39]. A minimum quality score of 25 with sequence length of 150 bp was employed and any reads below these levels were discarded. Taxonomy was assigned using BLAST [37] referenced against the SILVA SSURef database (version 111) [38] and OTU alignment was carried using PyNAST Phylogenetic tree [39]. Alpha diversity indices were calculated using QIIME.

#### Statistical analysis

The statistical analysis was carried out on SPSS v18. The independent *t* test was employed to assess for statistically significant differences between groups, with *p* < 0.05 considered significant. The Benjamini–Hochberg false discovery rate procedure was applied to sequencing data with *Q* set at 20%.

## Results

### Dietary fibre analysis

The pre-simulated digested CE was found to contain 60% soluble dietary fiber and 0.5% insoluble dietary fiber.

### Effects on bacterial composition as assessed by 454 pyrosequencing

**Fig. 1:**
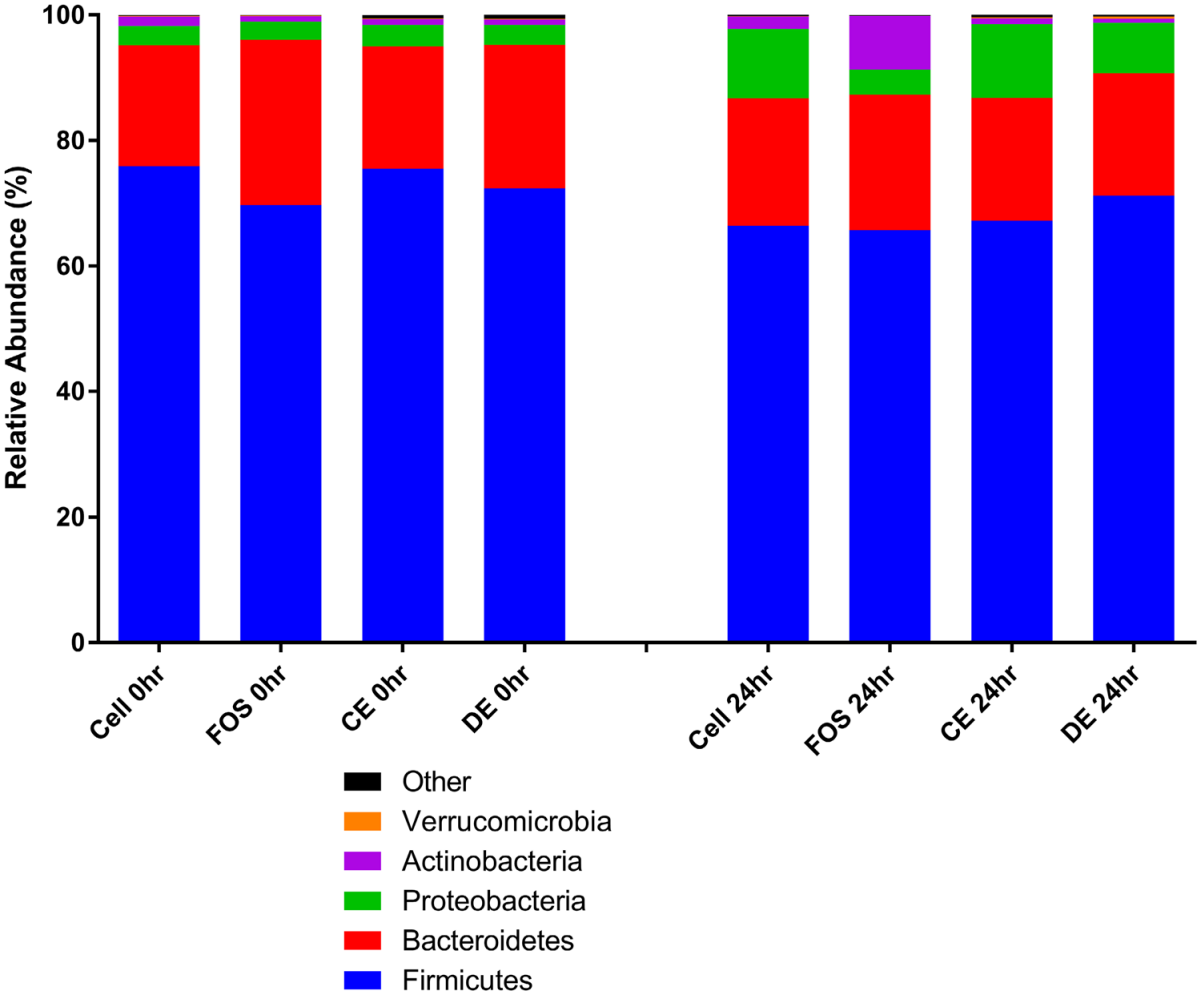
Mean relative abundances of the five most abundant bacterial phyla of cellulose (Cell), fructooligosaccharides (FOS), *Laminaria digitata* crude polysaccharide-rich extract (CE)- and *Laminaria digitata*-depolymerized polysaccharide-rich extract (DE) (1%)-treated fecal fermentation vessels (*n* = 3) at 0 and 24 h. Bacterial composition was assessed by 16S rDNA sequencing using 454 FLX technology and bacteria were assigned to their Phyla. The remaining Phyla were combined and represented within the group “Other”

**Fig. 2:**
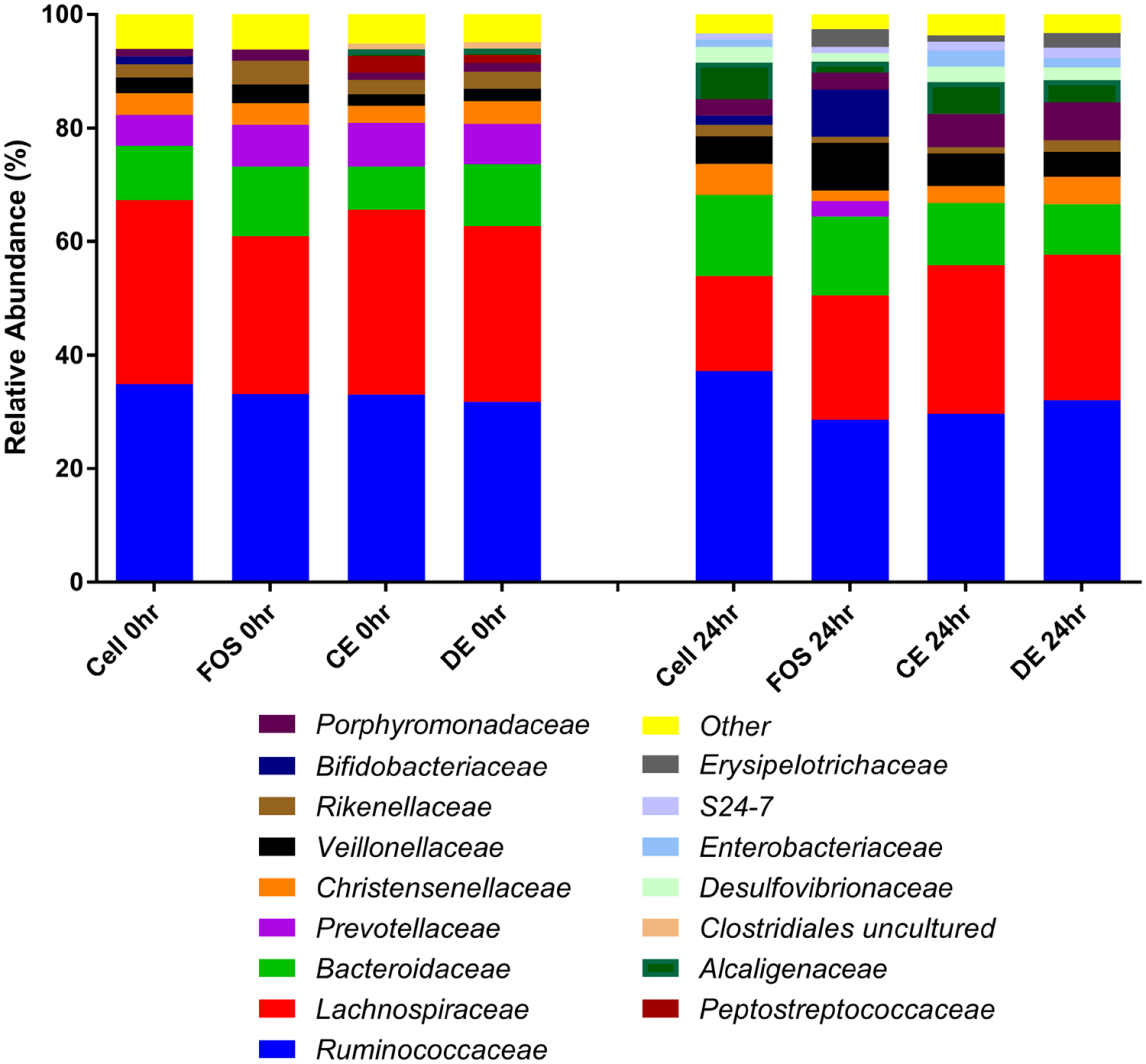
Mean relative abundances of bacterial families of cellulose (Cell), fructooligosaccharides (FOS), *Laminaria digitata* crude polysaccharide-rich extract (CE)- and *Laminaria digitata*-depolymerized polysaccharide-rich extract (DE) (1%)-treated fecal fermentation vessels (*n* = 3) at 0 and 24 h. Bacterial composition was assessed by 16S rDNA sequencing using 454 FLX technology and bacteria were assigned to their family. The figure represents the 15 most abundant families and any remaining families were combined and represented as other

**Fig. 3:**
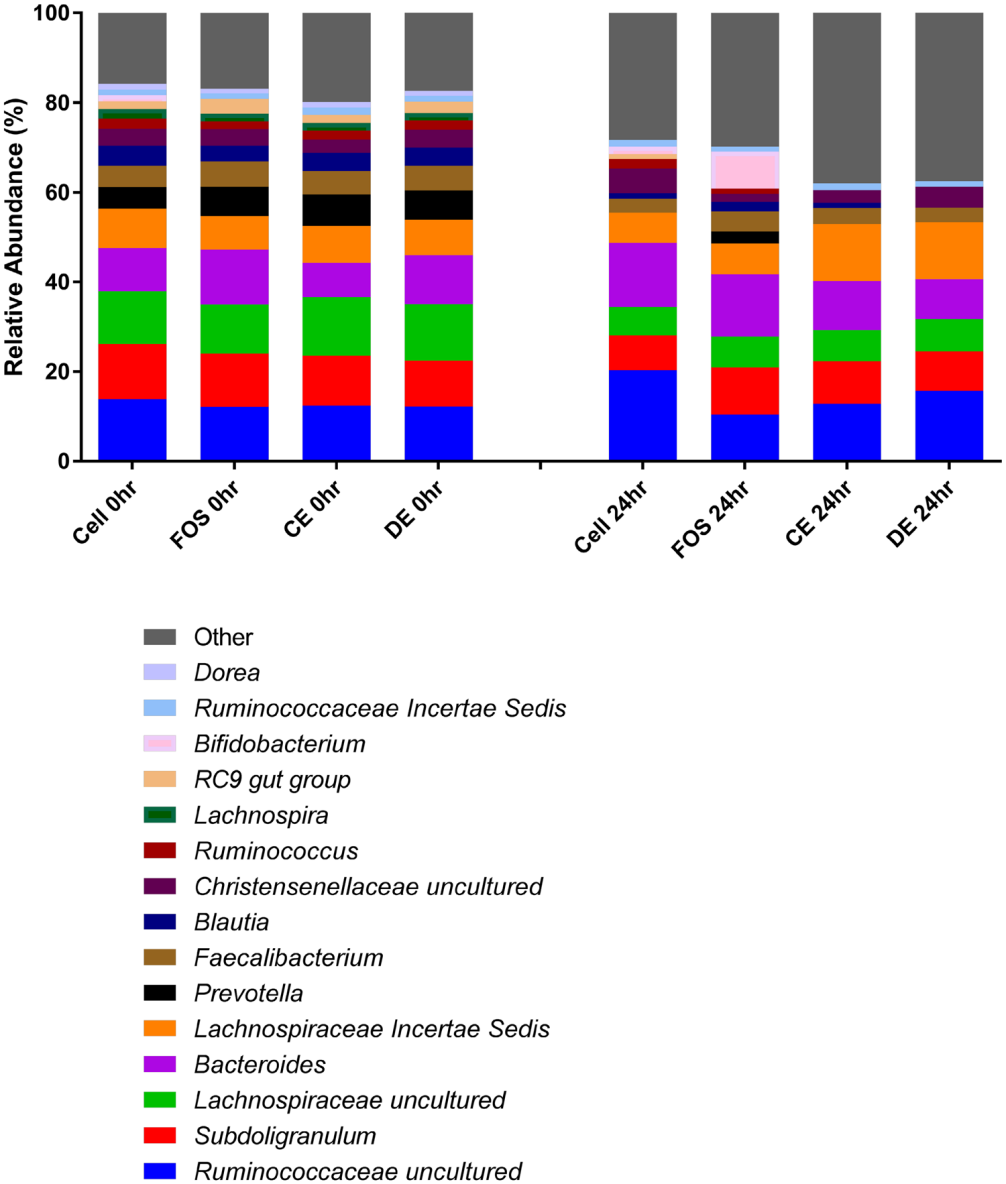
Mean relative abundances of bacterial genera of cellulose (Cell), fructooligosaccharides (FOS), *Laminaria digitata* crude polysaccharide-rich extract (CE)- and *Laminaria digitata*-depolymerized polysaccharide-rich extract (DE) (1%)-treated fecal fermentation vessels (*n* = 3) at 0 and 24 h. Bacterial composition was assessed by 16S rDNA sequencing using 454 FLX technology and bacteria were assigned to their genera. The figure represented the 15 most abundant genera and any remaining genera were combined and represented as other

All treatment vessels (Cellulose, FOS, CE and DE) at baseline (0 h) had similar proportions of phyla with Firmicutes the most abundant (69.67–75.881%) followed by Bacteroidetes (19.257–26.358%), Proteobacteria (2.881–3.449%) and Actinobacteria (0.873–1.499%) (Fig. 1). Other Phyla such as Cyanobacteria, Fibrobacteres, Fusobacteria, Lentisphaerae, Tenericutes, Verrucomicrobia, RF3 and bacteria unassigned to phyla (no blast hit) were lower in abundance. Figures 1, 2 and 3 show a graphical representation of the taxa at the Phylum, Family and Genus levels at time 0 and after 24 h of fermentation with the test substrates (Fig. 1—Phylum; Fig. 2—Family; Fig. 3—Genus).

**Fig. 4:**
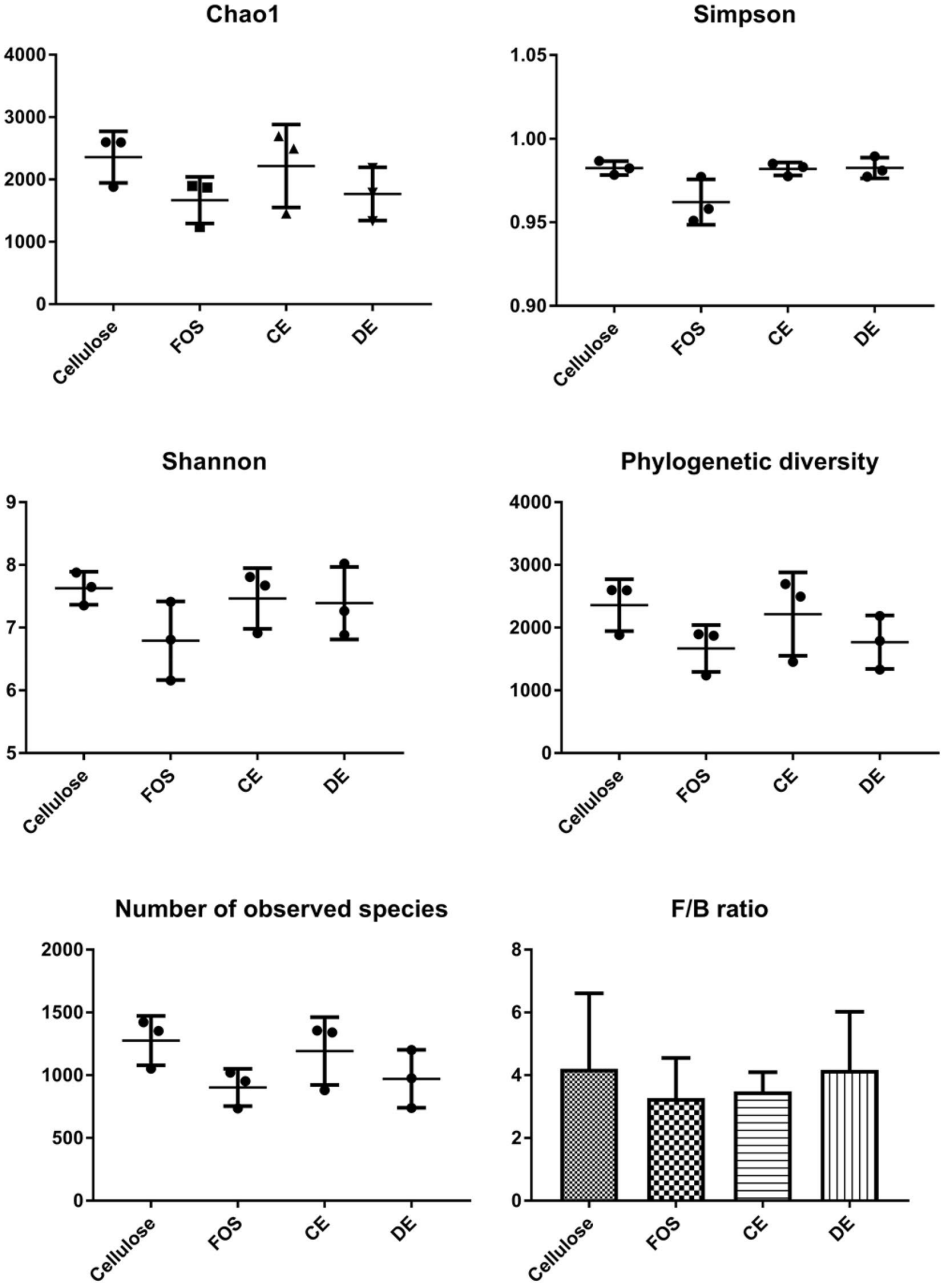
Mean alpha diversity indices and firmicutes:bacteroidetes ratios of cellulose (Cell), fructooligosaccharides (FOS), *Laminaria digitata* crude polysaccharide-rich extract (CE) and *Laminaria digitata*-depolymerized polysaccharide-rich extract (DE) (1%)-treated fecal fermentation vessels (*n* = 3) after 24 h. Bacterial composition was assessed by 16S rDNA sequencing using 454 FLX technology and alpha diversity indices were calculated using QIIME

There were no significant differences between any test compounds and cellulose for the alpha diversity indices Chao1, Simpson, Shannon, phylogenetic diversity and number of observed species after 24 h (Fig. 4). There were no significant effects of any test compounds on Firmicutes to Bacteroidetes ratios after 24 h of fermentation (Fig. 4).

#### Crude extract (CE) (Table 1)

**Table 1:** Significant differences induced by crude polysaccharide-rich extract (CE) treatment on bacterial Phyla, Families and Genera

	0 h		24 h		∆		Effect	*P*
	Cellulose (%)	CE (%)	Cellulose (%)	CE (%)	Cellulose	CE		
Phylum								
Fibrobacteres	0 (0)	0.009 (0.006)	0.04 (0.01)	0.012 (0.009)	+ 0.04 (0.005)	+ 0.003 (0.005)	**↓**	0.026
Family								
*Porphyromonadaceae*	1.305 (0.176)	1.232 (0.175)	2.856 (0.703)	5.941 (0.546)	+ 1.551 (0.813)	+ 4.709 (0.713)	**↑**	0.043
*Fibrobacteraceae*	0 (0)	0.009 (0.006)	0.04 (0.01)	0.012 (0.009)	+ 0.04 (0.005)	+ 0.003 (0.005)	**↓**	0.026
*Streptococcaceae*	0.067 (0.043)	0.06 (0.004)	0.097 (0.056)	0.028 (0.016)	+ 0.03 (0.014)	− 0.033 (0.012)	**↓**	0.025
*Lachnospiraceae*	32.303 (2.438)	32.55 (1.878)	16.718 (0.458)	26.182 (2.781)	− 15.585 (1.992)	− 6.368 (1.03)	**↑**	0.015
Genus								
*Parabacteroides*	0.488 (0.152)	0.511 (0.05)	1.293 (0.35)	5.085 (0.674)	+ 0.805 (0.255)	+ 4.574 (0.633)	**↑**	0.005
*Fibrobacter*	0 (0)	0.009 (0.006)	0.04 (0.01)	0.012 (0.009)	+ 0.04 (0.005)	+ 0.003 (0.005)	**↓**	0.026
*Streptococcus*	0.06 (0.041)	0.06 (0.004)	0.097 (0.056)	0.028 (0.016)	+ 0.037 (0.015)	− 0.033 (0.012)	**↓**	0.022
*Ruminococcus*	2.235 (0.983)	2.058 (0.524)	2.204 (1.291)	0.675 (0.377)	− 0.032 (0.369)	− 1.383 (0.148)	**↓**	0.027
*Dialister*	0.589 (0.107)	0.504 (0.169)	0.132 (0.084)	0.463 (0.136)	− 0.457 (0.037)	− 0.042 (0.062)	**↑**	0.005
*γ* B38*UC*	0.019 (0.009)	0.007 (0.007)	0.029 (0.01)	0.041 (0.003)	+ 0.01 (0.008)	+ 0.034 (0.004)	**↑**	0.045

An overview of the significant changes in the relative abundance of the Phyla, Family and Genus (as determined by 454 pyrosequencing of the 16S rDNA region) of the batch culture fermentate following a 24-h incubation with 1% (w/v) CE or cellulose under anaerobic conditions at 37 °C. Significant differences of delta change values (∆) (difference 0 and 24 h) between CE and cellulose were determined using independent *t* tests (*n* = 3). Data presented are mean (SEM)

The Phyla Fibrobacteres was significantly reduced in CE-treated vessels in comparison to cellulose-treated control vessels (*p* = 0.026, *q* = 0.018) (see Table 1). At the family level, *Porphyromonadaceae* (*p* = 0.043, *q* = 0.014) and *Lachnospiraceae* (*p* = 0.015, *q* = 0.004) were significantly increased compared to cellulose. *Porphyromonadaceae* was the fourth most dominant bacterial family after 24 h of CE treatment as opposed to the seventh in cellulose-treated vessels. In contrast, *Fibrobacteraceae* (*p* = 0.026, *q* = 0.01) and *Streptococcaceae* (*p* = 0.025, *q* = 0.007) were both reduced with CE treatment (see Table 1).

The increases in *Porphyromonadaceae* could be attributed to the stimulation of *Parabacteroides* species which increased significantly compared to control vessels (+ 4.754% vs. + 0.805%; *p* = 0.005, *q* = 0.003) (Table 1). There was a modest but significant increase in an uncultured genera belonging to the *B38* Proteobacteria lineage (*p* = 0.045, *q* = 0.008), and also an increase in *Dialister* species compared to cellulose-treated vessels was noted (*p* = 0.005, *q* = 0.001). *Ruminococcus* (*p* = 0.027, *q* = 0.007), *Streptococcus* (*p* = 0.022, *q* = 0.004) and *Fibrobacter* (*p* = 0.026, *q* = 0.005) species were all reduced after 24 h of fermentation of CE compared to cellulose-treated vessels. There was no significant increase of any *Lachnospiraceae* genera, suggesting that the increases in this family occurred division wide.

#### Depolymerized extract (DE) (Table 2)

**Table 2:** Significant differences induced by depolymerized crude polysaccharide-rich extract (DE) on bacterial Phyla, Families and Genera

	0 h		24 h		∆		Effect	*P*
	Cellulose (%)	DE (%)	Cellulose (%)	DE (%)	Cellulose	DE		
Phylum								
Actinobacteria	1.499 (0.299)	0.873 (0.218)	1.955 (0.284)	0.607 (0.13)	+ 0.456 (0.068)	− 0.266 (0.171)	**↓**	0.017
Family								
*Lachnospiraceae*	32.303 (2.438)	30.968 (1.478)	16.718 (0.458)	25.724 (3.06)	− 15.585 (1.992)	− 5.244 (2.791)	**↑**	0.039
*Alcaligenaceae*	0.636 (0.088)	1.071 (0.142)	6.444 (0.569)	3.864 (0.54)	+ 5.807 (2.793)	+ 2.793 (0.682)	**↓**	0.030
Genus								
*Parabacteroides*	0.488 (0.15)	0.618 (0.206)	1.293 (0.35)	5.64 (1.234)	+ 0.805 (0.255)	+ 5.022 (1.039)	**↑**	0.017
*Lachnospiraceae uc*.	0 (0)	0.007 (0.003)	0.003 (0.003)	0.046 (0.003)	+ 0.003 (0.003)	+ 0.039 (0.005)	**↑**	0.003
*Peptostreptococcaceae IS*	0.846 (0.048)	1.299 (0.074)	0.32 (0.085)	0.244 (0.074)	− 0.526 (0.051)	− 1.056 (0.147)	**↓**	0.027
*Dialister*	0.589 (1.234)	0.418 (0.107)	0.132 (0.084)	0.277 (0.104)	− 0.47 (0.037)	− 0.141 (0.054)	**↑**	0.008

An overview of the significant changes in the relative abundance of the Phyla, Family and Genus (as determined by 454 pyrosequencing of the 16S rDNA region) of the batch culture fermentate following a 24-h incubation with 1% (w/v) DE or cellulose under anaerobic conditions at 37 °C. Significant differences of delta change values (∆) (difference 0 and 24 h) between DE and cellulose were determined using independent *t* tests (*n* = 3). Data presented are mean (SEM)

In common with CE extract, DE extract had a significant effect on the relative abundance of the genus *Parabacteroides* (*p* = 0.017), with DE treatment significantly increasing the relative abundance compared to cellulose (Table 2). DE treatment also had an effect on several families and genera within the Clostridiales order. The relative abundance of the *Lachnospiraceae* family was significantly higher after 24 h of fermentation when compared to cellulose (*p* = 0.039, *q* = 0.007) and within this family there were significant increases in an uncharacterized bacterium with DE treatment compared to cellulose (*p* = 0.003, *q* = 0.001), although this was a less abundant genus that was not detected at 0 h in the cellulose vessel. A significant decrease in the relative abundance of the *Peptostreptococcaceae Incertae Sedis* family was observed with DE treatment. It should be noted that the relative abundances of the *Peptostreptococcaceae Incertae Sedis* family were statistically different at 0 h between DE and cellulose. As with the CE treatment, the relative abundance of *Dialister* was significantly higher with DE treatment compared to cellulose (*p* = 0.008, *q* = 0.003). The relative abundance of the *Alcaligenaceae* family belonging to the *Proteobacteria* Phyla was significantly reduced compared to cellulose (*p* = 0.03, *q* = 0.004).

#### Fructooligosaccharides (Table 3)

**Table 3:** Significant differences induced by FOS treatment on bacterial Phyla, Families and Genera

	0 h		24 h		∆		Effect	*P *
	Cellulose (%)	FOS (%)	Cellulose (%)	FOS (%)	Cellulose	FOS		
Phylum								
Proteobacteria	3.11 (0.312)	2.881 (0.279)	11.082 (1.226)	3.968 (1.628)	+ 7.972 (1.368)	+ 1.087 (1.904)	↓	0.043
Family								
*Streptococcaceae*	0.067 (0.043)	0.129 (0.045)	0.097 (0.056)	0.003 (0.003)	+ 0.03 (0.014)	− 0.126 (0.043)	↓	0.025
*Clostridium XIII IS*	0.032 (0.013)	0.011 (0.011)	0.069 (0.067)	0.227 (0.063)	+ 0.037 (0.016)	+ 0.216 (0.053)	↑	0.032
*Lachnospiraceae*	32.303 (2.438)	27.771 (0.886)	16.718 (0.485)	21.909 (1.271)	− 15.585 (1.99)	− 5.862 (0.387)	↑	0.009
*Alcaligenaceae*	0.636 (0.088)	0.988 (0.262)	6.444 (0.569)	1.959 (0.612)	+ 5.807 (0.612)	+ 0.971 (0.848)	↓	0.010
Genus								
*Butyricimonas*	0.072 (0.029)	0.187 (0.046)	0.288 (0.081)	0.079 (0.023)	+ 0.217 (0.094)	− 0.109 (0.032)	↓	0.030
*Parabacteroides*	0.488 (0.152)	0.735 (0.11)	1.293 (0.35)	0.579 (0.11)	+ 0.805 (0.255)	− 0.155 (0.084)	↓	0.023
*Streptococcus*	0.06 (0.041)	0.129 (0.045)	0.097 (0.056)	0.003 (0.003)	+ 0.037 (0.015)	− 0.125 (0.043)	↓	0.023
*Christensenella*	0.058 (0.019)	0.041 (0.015)	0.094 (0.027)	0.033 (0.005)	+ 0.037 (0.009)	− 0.007 (0.01)	↓	0.033
*Clostridium XIII IS UC*	0.012 (0.012)	0.008 (0.008)	0.023 (0.006)	0.212 (0.052)	+ 0.013 (0.016)	+ 0.204 (0.045)	↑	0.016
*Flavonifractor*	0.085 (0.017)	0.079 (0.004)	0.282 (0.032)	0.082 (0.006)	+ 0.197 (0.046)	+ 0.003 (0.008)	↓	0.047
*Sutterella*	0.451 (0.046)	0.832 (0.302)	5.304 (1.0)	1.569 (0.471)	+ 4.85 (0.989)	+ 0.737 (0.772)	↓	0.030
*Cronobacter*	0 (0)	0.003 (0.003)	0.116 (0.023)	0.02 (0.016)	+ 0.116 (0.023)	+ 0.017 (0.012)	↓	0.019

An overview of the significant changes in the relative abundance of the Phyla, Family and Genus (as determined by 454 pyrosequencing of the 16S rDNA region) of the batch culture fermentate following a 24-h incubation with 1% (w/v) FOS or cellulose under anaerobic conditions at 37 °C. Significant differences of delta change values (∆) (difference 0 and 24 h) between FOS and cellulose were determined using independent *t* tests (*n* = 3). Data presented are mean (SEM)

FOS-fermented vessels had significantly lower proportions of Proteobacteria (*p* = 0.043, *q* = 0.018), with significantly lower representations of *Alcaligenaceae* (*p* = 0.01, *q* = 0.007) compared to cellulose. The reduction in *Alcaligenaceae* abundance was mostly owing to reductions in the relative abundance of *Sutterella* (*p* = 0.03, *q* = 0.009) compared to cellulose. There was a significant reduction in the relative abundance of the gamma Proteobacteria genus *Cronobacter* (*p* = 0.019, *q* = 0.004). FOS fermentation significantly decreased the relative abundance of two Bacteroidetes genera belonging to the *Porphyromonadaceae* family, *Butyricimonas* (*p* = 0.03, *q* = 0.008) and *Parabacteroides* (*p* = 0.023, *q* = 0.007). Within the Firmicutes phylum, FOS treatment resulted in a significant reduction in *Streptococcus* (*p* = 0.023, *q* = 0.005), *Christensenella* (*p* = 0.033, *q* = 0.01) and *Flavonifractor* (*p* = 0.047, *q* = 0.012) and an increase in the relative abundance of *Lachnospiraceae* (*p* = 0.009, *q* = 0.004) and a poorly understood family (*p* = 0.032, *q* = 0.014) and genus (*p* = 0.016, *q* = 0.003) belonging to the Clostridiales Family XIII, when compared to cellulose.

### Effects on culture-dependent *Lactobacillus* and *Bifidobacteria* populations

Neither CE nor DE stimulated cultivable *Lactobacillus* or *Bifidobacteria* species numbers at any time point. FOS was found to significantly (*p* = 0.044) increase cultivable *Bifidobacteria* populations after 10 h compared to cellulose control.

### Effects on short-chain fatty acid production

#### Crude extract (CE) (Table 4)

**Table 4:** Fatty acid concentrations produced during batch culture fermentations

	Cellulose		FOS			CE			DE		
	Mean	SEM	Mean	SEM	*P* value	Mean	SEM	*P* value	Mean	SEM	*P* value
Acetic acid											
5	11.89	1.195	39.41	4.810	0.005	16.80	2.253	0.126	19.55	3.386	0.100
10	16.10	1.068	45.95	4.080	0.002	30.35	1.183	0.001	42.10	5.413	0.036
24	17.95	0.101	51.36	4.492	0.002	39.71	2.122	0.009	48.65	2.538	0.007
36	19.48	0.581	53.70	4.762	0.002	45.49	1.929	0.000	57.62	8.973	0.013
48	20.12	0.596	55.88	4.512	0.001	54.11	11.674	0.044	54.32	3.772	0.001
Propanoic acid											
5	2.75	0.196	11.06	2.632	0.035	4.53	0.177	0.003	6.06	0.509	0.004
10	3.63	0.228	15.13	3.087	0.021	10.68	1.382	0.007	17.89	2.303	0.004
24	4.49	0.317	17.25	2.970	0.013	15.49	1.852	0.004	20.69	1.984	0.001
36	5.11	0.149	18.26	3.107	0.013	17.80	1.326	0.010	24.39	3.999	0.009
48	5.42	0.287	19.33	3.525	0.017	21.59	5.527	0.043	23.02	2.104	0.001
Butyric acid											
5	3.37	0.109	13.09	0.565	0.000	5.43	0.829	0.070	5.09	0.755	0.089
10	4.89	0.236	22.25	2.144	0.001	11.04	1.868	0.031	9.80	1.628	0.041
24	6.65	0.148	26.44	2.867	0.002	16.01	2.090	0.011	12.42	1.467	0.017
36	7.78	0.229	28.67	2.982	0.002	19.33	2.760	0.014	15.10	1.721	0.049
48	8.50	0.195	30.69	3.036	0.002	22.92	0.702	0.000	15.18	1.851	0.023
Total SCFA											
5	18.98	1.515	63.92	2.837	0.000	27.84	3.692	0.091	32.62	5.960	0.091
10	26.62	1.500	84.88	1.544	0.000	56.14	3.701	0.002	72.99	10.163	0.011
24	32.76	1.118	98.69	0.873	0.000	75.76	1.271	0.000	88.18	8.139	0.009
36	36.84	1.638	105.56	1.450	0.000	89.46	3.577	0.000	104.72	16.786	0.016
48	39.13	1.366	112.04	2.861	0.000	107.79	17.710	0.018	99.98	6.970	0.001
BCFA											
5	0.99	0.347	0.44	0.239	0.260	0.96	0.505	0.967	1.53	1.022	0.645
10	1.93	0.362	1.43	0.659	0.539	3.80	2.699	0.531	2.52	1.381	0.703
24	3.31	0.754	2.98	0.840	0.781	3.64	0.745	0.774	5.05	2.785	0.602
36	4.05	0.737	4.07	0.857	0.988	5.24	0.987	0.389	6.29	2.395	0.424
48	4.65	0.596	5.14	0.765	0.639	7.37	0.951	0.072	6.32	1.464	0.350

A table listing the concentrations of short-chain fatty acids in the effluent of batch culture vessels after incubation with cellulose, FOS, CE and DE after 5, 10, 24. 36 and 48 h. Samples were taken at the various time points, centrifuged, and the SCFA were quantified using a GC-FID. Significant differences in the individual SCFA, total SFCA and total BCFA between cellulose and each treatment (FOS, DE, CE) at each time point was determined using an independent *t* test. *p < 0.05 (*n* = 3)

**Fig. 5:**
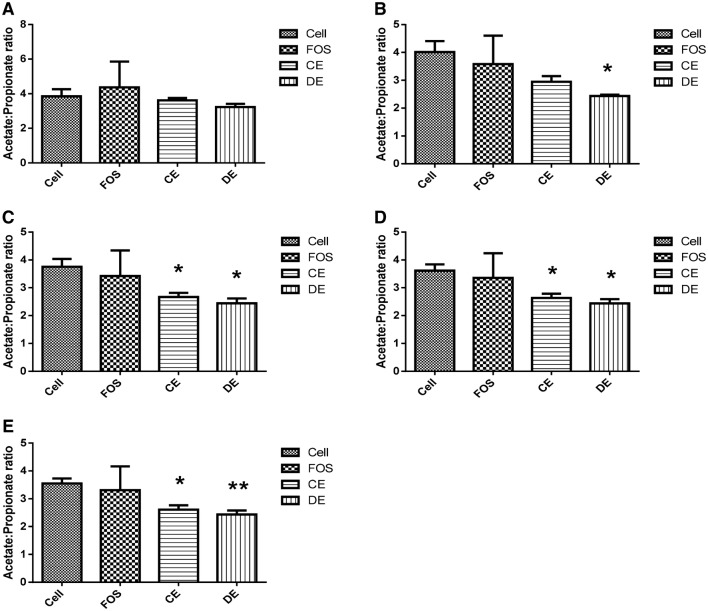
The effects of 1% (w/v) cellulose (Cell), fructooligosaccharides (FOS), crude polysaccharide-rich extract (CE); and depolymerized crude polysaccharide-rich extract (DE), on the acetate to propionate ratio of batch culture fermentate after 5 (**a**), 10 (**b**), 24 (**c**), 36 (**d**) and 48 (**e**) h of incubation under anaerobic conditions at 37 °C. SCFA concentrations of each supernatant were assessed using a GC-FID. Significant differences in the acetate to propionate ratio between cellulose and each treatment (FOS, DE, CE) at each time point were determined using an independent *t* test. *p < 0.05 (*n* = 3)

Within 5 h of fermentation of the CE, propionic acid concentrations were significantly increased (*p* = 0.003), and remained significantly higher over all subsequent time points in comparison to the cellulose control. Propionic acid concentrations in the CE-treated vessels were similar to that of the FOS-treated vessels for all time points assessed. After 10 h of CE fermentation, acetic acid (*p* = 0.001), butyric acid (*p* = 0.031), and total SCFA (*p* = 0.002) concentrations were also significantly higher than cellulose. Concentrations of acetic acid, butyric acid, and total SCFA were significantly higher with CE treatment compared to cellulose for all subsequent time points. The acetate:propionate ratio was significantly lower than the cellulose control following 24, 36 and 48 h of fermentation (*p* < 0.05) (Fig. 5). There were no significant differences in the concentration of valeric acid, hexanoic acid, the individual branched-chain SCFAs iso-butyric acid and iso-valeric acid, nor total branched SCFA and between CE and cellulose at any time point.

When compared to FOS, CE fermentation produced significantly lower levels of acetic acid after 5 (*p* = 0.013) and 10 h (*p* = 0.021) of fermentation with no significant difference observed between CE and FOS after 24, 36 and 48 h of fermentation. Butyric acid concentrations were significantly lower with CE fermentation after 5 (*p* = 0.002), 10 (*p* = 0.017) and 24 (*p* = 0.042) hours compared to FOS, but no significant difference was noted after 36 and 48 h of fermentation. Total SCFA concentrations were significantly lower after 5, 10, 24 and 36 h with CE compared to FOS, yet total SCFA concentrations were similar after 48 h of fermentation. Propionic acid, iso-butyric acid, valeric acid, iso-valeric acid and hexanoic acid concentrations with CE treatment were similar to FOS over all time points assessed, with no significant differences noted.

#### Depolymerized extract (DE) (Table 4)

Within 5 h of fermentation of the DE extract, the concentration of propionic acid was significantly higher than in cellulose vessels (*p* = 0.004) and the concentration of propionic acid was significantly higher compared to cellulose for all the subsequent time points. Propionic acid concentrations were similar to FOS at all time points. The concentrations of acetic acid, propionic acid, butyric acid, and total SCFA were significantly higher with DE treatment compared to cellulose after 10 h and for all subsequent time points. The acetate:propionate ratio was shown to be significantly lower than the cellulose control following 10, 24, 36 and 48 h (*p* < 0.05) (Fig. 5). There were no significant differences in the concentration of total branched SCFA, the individual branched-chain SCFAs iso-butyric acid and iso-valeric acid or valeric acid and hexanoic acid SCFAs between CE and cellulose at any time point. Compared to FOS, acetic acid and total SCFA concentrations were significantly lower after 5 h of fermentation (*p* = 0.028 and *p* = 0.009, respectively), but not after 10, 24, 36 and 48 h of DE fermentation. Butyric acid concentrations were significantly lower compared to FOS for all the time points assessed.

#### Fructooligosaccharides (FOS) (Table 4)

Within 5 h of incubation, FOS was observed to significantly increase the concentration of acetic acid (*p* = 0.005), propionic acid (*p* = 0.035), butyric acid (*p* < 0.001), and total SCFA (*p* < 0.001) compared to cellulose. The concentrations were significantly higher for all subsequent time points. There was no difference in branched-chain SCFA with FOS fermentation compared to cellulose.

## Discussion

This study has provided clear indications that crude and depolymerized non-digestible components of *L. digitata* (CE and DE) are fermented by human fecal bacterial populations. This was evidenced by significant increases in acetate, butyrate and total short-chain fatty acids after 10-h fermentation with CE, as well as DE compared to cellulose (Table 4), and was shown to continue for the majority of the remaining incubation time in the experiment. The culture-dependent microbial analysis showed that neither CE nor DE stimulated *Lactobacilli* or *Bifidobacteria* populations. The culture-independent sequencing of the 16S region revealed a number of changes at the family and genus level when compared to cellulose, including the stimulation of *Parabacteroides, Dialister* and *Lachnospiraceae* populations, each of which have been shown to be reduced in IBD-associated dysbiosis, with *Lachnospiraceae* also suggested to be protective against colon cancer [40–42].

The ability of the microbiota to utilize the seaweed fiber components as an energy source is primarily dictated by the presence of enzymes which can hydrolyze the various glycosidic bonds throughout the fiber structure [43]. There are numerous in vitro human fecal batch culture experiments previously carried out on each individual seaweed fiber (alginate/laminarin/fucoidan), and these studies have demonstrated that both alginate and laminarin fibers can be efficiently fermented by the human microbiota [24, 25, 31–35, 44, 45]. These observations are supported by previous reports that *Bacteroides* species from the human microbiota possess the hydrolytic enzymes, β-glucanase and alginate lyase, which are able to breakdown the linkages that make up laminarin and alginate, respectively, and have been noted in both Asian and Western populations [31, 46–48]. There is evidence from a murine study to show that fucoidan can also modulate the composition and metabolic activity of the gut microbiota. Dietary supplementation of a 50-kDa fucoidan fraction in combination with the chemotherapeutic drug associated with gut mucosal damage, cyclophosphamide was found to increase *Coprococcus, Rikenella*, and *Butyricicoccus* species and increase total SCFA, propionate and butyrate concentrations in feces. Whether the effects on gut microbiota composition and metabolic activity are through fucoidan fermentation or through protection against mucosal damage from cyclophosphamide supplementation remains to be elucidated. The degradation of polysaccharides by the microbiota can ultimately lead to metabolism/cross metabolism of the released monomers to produce a range of microbial products, including short-chain fatty acids amongst others [49]. The failure to degrade fucoidan may limit its potential impact on the microbiota and thus any noted changes would be independent of its catabolism and fermentation.

The current study undertaken did not characterize the polysaccharide structural composition of either CE or DE; however, the increasing SCFA production following incubation with the human fecal matter in the fermenter would suggest that bacteria present in the human microbiota possess the enzymes capable of degrading at least some of the complex polysaccharides present in the seaweed extract. The fermentation of both the extracts resulted in significant increases in propionic acid compared to cellulose and both the extracts were similar to the prebiotic FOS with respect to propionic acid production after 5 h of fermentation and for all subsequent time points. Acetic acid, butyric acid, and total SCFA concentrations were significantly higher with both CE and DE compared to cellulose after 10 h of fermentation. CE fermentation resulted in similar amounts of acetic acid and butyric acid as FOS after 24 and 36 h of fermentation, respectively, and for subsequent time points. DE fermentation resulted in increases in acetic acid and total SCFA which were comparable with those produced by FOS after 10 h of fermentation and for all subsequent time points. Both extracts were shown to significantly reduce the acetate to propionate ratio after 24 h, which persisted for the remainder of the experiment. This could hold significance in light of recent research that has implicated a reduced acetate to propionate ratio with lipogenesis [50, 51], as well as emerging evidence to implicate a role for colonic propionate in the prevention of weight gain in overweight adult humans [52].

The fermentation patterns noted in the current study are comparable to previous studies that have investigated the fermentability of individual seaweed polysaccharides in both in vitro human fecal fermentation experiments and in vivo animal models [24, 25, 31–35, 44] and demonstrated that brown seaweed-derived polysaccharides increased the production of acetic acid, propionic acid, butyric acid as well as total SCFA. The crude polysaccharide extracts (CE, DE) used in the current study exhibited a lag effect, with the production of most of the SCFA occurring after 10 h and not 5 h. This lag effect has previously been reported with alginate [44], which is known to be a major polysaccharide component present in *L. digitata* [53]. This lag effect of fermentation may be attributed to the adaption of the fecal bacterial species to a novel carbon source [54]. The higher production of propionate noted during DE fermentation compared to CE after 5 h is likely owing to the lower molecular weight polysaccharides (> 1 kDa), with alginate a probable candidate [31, 34, 44]. The CE treatment had significantly higher concentrations of butyrate after 48 h of fermentation compared to DE, suggesting a more sustained, slower fermentation profile which would be expected with more complex, higher molecular weight polysaccharides [55]. Previous batch fermentation studies have demonstrated that laminarin and alginate individually increase SCFA concentrations during batch culture fermentations whilst fucoidan would appear to have limited impact [24, 44]. Furthermore, the fermentation properties of the complete fiber component of late-season-harvested *L. digitata* have been assessed with human fecal fermentation experiments [24, 25]. The first study [24] found that *L. digitata* fiber fermentation appeared to alter individual SCFA ratios as well as increase total SCFA concentrations with a similar lag period noted in this study. While the second study [25] did not assess effects on SCFA, the authors observed that laminarin was fermented after a 24-h incubation. Collectively, it is likely that the fermentation of both alginate and laminarin is contributing to the potential prebiotic effect of increasing both butyrate and propionate production observed in the current study.

Neither the CE nor DE extract stimulated cultivable *Lactobacillus* or *Bifidobacteria* species, and this lack of effect was also reported in a study by Deville et al. [25]. In contrast, others have found a stimulating effect with alginate oligosaccharides on *Lactobacillus* and *Bifidobacteria*, both with in vitro human fecal fermentations and when fed to rats [56]. The size-exclusion filtration carried out on the extract used in this study may have removed these oligosaccharides (< 1 kDa) and thus could explain the lack of effect seen in this study. In the current study, FOS treatment was observed to significantly increase cultivable *Bifidobacteria* but not *Lactobacillus*, whereas another study has reported that *Lactobacillus* and not *Bifidobacteria* populations were increased with FOS [25]. The lack of consistency in the effects of certain fibers to stimulate specific bacterial genera from in vitro studies may be attributable, in part, to the limited study power owing to variability of the fecal microbial composition of the donors and highlights the problematic issues of using such models to investigate dietary-mediated changes to GM structure and composition.

Results from the 16S rRNA pyrosequencing identified that both the CE and DE extracts impacted mainly within the two dominant phyla, Bacteroidetes and Firmicutes. As previously discussed, the Bacteroidetes species have considerable metabolic plasticity owing to an extensive array of Carbohydrate-Active Enzymes (CAZymes) including seaweed-glycan-degrading enzymes [31, 57], which allow them to catabolize the wide range of polysaccharides present in the human diet. Therefore, it is not surprising that the polysaccharide-rich seaweed extracts were found to significantly increase genera belonging to this phylum. The ability of both extracts to significantly increase the relative abundance of bacteria from the genus *Parabacteroides* was also observed in another study in which laminarin was fed to rats [30]. The authors noted that the species *Parabacteroides distasonis* was only detected in the laminarin-supplemented rats and not alginate-supplemented or control-fed rats. This finding would strongly suggest that the *Parabacteroides* stimulating effect noted in the current study is likely to be attributable to laminarin. A recent study highlighted the potential immunomodulatory effect of *Parabacteroides distasonis* in DSS-induced colitis in mice [58], albeit more evidence to support its possible impact on health in humans is required. *Lachnospiraceae* were also found to be stimulated with both CE and DE extracts. *Lachnospiraceae* are well represented in the human gut microbiome and are known to degrade non-digestible plant polysaccharides from the diet into SCFAs such as acetate, propionate and butyrate [59, 60]. The increase in SCFA production observed with the extracts in this study could, in part, be attributed to bacteria belonging to this family. The observed depletion of *Lachnospiraceae* members in colon cancer has led to speculation that some of its members may confer protection against colon cancer through the production of butyrate, which is known to exert a range of anticancer activities [61]. The stimulating effect of DE on bacteria from the *Dialister* genus was also found to be inversely associated with IL-6 in humans who were fed a fiber-rich diet from whole grains [62]; however, some reductions in *Dialister species* have been reported in colon cancer and Crohn’s disease [63, 64].

The extracts in the current study were subjected to a simulated in vitro digestion followed by size-exclusion chromatography with a molecular weight cutoff of 1 kDa prior to use in the colon model. The simulated digestion used in the current study provided indications that digestion-resistant polysaccharides present within the CE and DE extract had the potential to be extensively fermented by the human microbiota, which corroborates previous studies which have demonstrated that isolated laminarin, fucoidan and alginate are resistant to simulated gastrointestinal digestion [45]. However, whilst the dialysis step in the simulated digestion removes contents unlikely to be available in the colon, it may also remove colon-available non-digestible low molecular weight oligosaccharides such as those derived from alginate and laminarin. This presents an additional limitation to this in vitro study.

## Conclusions

In conclusion, this study provides evidence that polysaccharide-rich extracts from *L. digitata* are both resistant to human digestive enzymes and are fermentable by human fecal bacterial populations. The demonstration that both extracts altered the metabolic activity of human fecal microbial populations in a way which might confer health benefits illustrates the potential of this seaweed extract to improve health and well-being. Further work is warranted to assess whether these changes in both bacterial composition and metabolic activity occur in vivo, and critically, whether such changes can confer health benefits to the host to clarify the potential of *L. digitata* as a source material for the crude extraction of fermentable polysaccharide fibers as a functional food ingredient.
